# Prediction of metaphase II oocytes according to different serum Anti-Müllerian hormone (AMH) levels in antagonist ICSI cycles

**DOI:** 10.5935/1518-0557.20160043

**Published:** 2016

**Authors:** Joyce B da Silva, Tatiana R Panaino, Maria A Tamm, Paloma Lira, Patricia C F Arêas, Ana C A Mancebo, Marcelo M de Souza, Roberto A Antunes, Maria do Carmo B de Souza

**Affiliations:** 1 Fertipraxis Reproduction Center, Rio de Janeiro, RJ, Brazil

**Keywords:** Anti-Müllerian hormone (AMH), metaphase II oocytes, ovarian response, controlled ovarian stimulation, IVF outcome

## Abstract

**Objective:**

This paper aims to assess a qualitative aspect of ovarian response in terms of metaphase II oocytes according to different serum Anti-Müllerian hormone levels in antagonist ICSI cycles. A prediction index might contribute to the individualization of care.

**Methods:**

This observational study looked into 287 antagonist ICSI cycles carried out with patients treated in a single center between January of 2012 and January of 2016. Serum AMH and subgroup analyses were performed based on five AMH ranges (≤ 0.3 ng/mL;> 0.3 and ≤ 0.7 ng/mL; > 0.7 and ≤ 1.0 ng/mL; > 1.0 and < 3.0 ng/mL; ≥ 3.0 ng/mL). The variables analyzed included patient age; serum FSH and antral follicle count at the start of the cycle; number of stimulation days and number follicles ≥ 15 mm on hCG day; number of oocytes retrieved and number of metaphase II oocytes.

**Results:**

AMH is a better predictor of ovarian response to controlled ovarian stimulation than AFC or serum FSH, while age is an independent marker. AMH levels ≤0.70 (patients with poor prognosis) were observed in 140 patients (48.7%). Patients within this AMH level range accounted for 92% of the 24 failed cycles (cancelled cycles, no oocytes or immature oocytes retrieved).

**Conclusion:**

AMH predicts the quality of ovarian response to stimulation, regardless of patient age. Women with AMH levels ≥1.0 and ≤3.0 ng/mL are probably normal responders with good prognosis. Clinical application relies on the examination of the data from each individual center and on the establishment of correlations between AMH levels and ovarian response in the form of metaphase II oocytes.

## INTRODUCTION

One of the most difficult aspects of individualizing assisted reproduction care is the identification of the actual ovarian reserve and counseling patients with very low chances of achieving pregnancy (Lee *et al*., 2011). Ovarian reserve tests provide knowledge of a patient's possible response, permitting the management of the appropriate gonadotropin dosages (Fleming *et al*., 2013). Several parameters have been postulated as predictors of ovarian response, including serum markers (FSH, inhibin B, 17-ß-estradiol, and anti-Müllerian hormone) and ultrasound variables (ovarian volume, measurement of antral follicles and ovarian stromal blood flow). Even after adjustment for chronological age, antral follicle count (AFC) and serum AMH correlate with ovarian primordial follicle number (Hansen *et al*., 2011; Aydin *et al*., 2015).

The release of AMH from ovarian granulosa cells leads to measurable serum levels, which are proportional to the number of developing follicles in the ovaries and appear to regulate early follicle development (La Marca *et al*., 2005). AMH is expressed in small and large pre antral follicles and in small antral follicles, the latter of which one of the main contributors to AMH serum levels. Initial recruitment of ovarian follicles is a continuous process, whereas cyclic recruitment is driven by a rise in FSH serum levels at the end of a previous menstrual cycle (Broekmans *et al*., 2008). The expression of the AMH receptor in granulosa cells suggests that it may play a role in ovarian physiology (La Marca & Volpe, 2006), and the main physiological role of AMH in the ovary seems to be the inhibition of the early stages of follicular development (Visser & Themmen, 2005). Detectable at birth, AMH levels rise in the weeks after birth to reach a peak after puberty (Bergadá *et al*., 2006; Guibourdenche *et al*., 2003). In prepubertal girls, AMH levels seem to be low with a tendency to rise towards the onset of puberty, and the hormone continues to be expressed in the growing follicles in the ovary until they have reached the size and differentiation state at which they are to be selected for dominance by the action of pituitary FSH. In adult women, serum AMH levels have been shown to decline gradually with age, as a sign of follicular exhaustion, becoming undetectable in menopause (Van Rooij *et al*., 2005).

AMH seems to exhibit a fairly stable consistent pattern of expression during the menstrual cycle, making it an attractive determinant of ovarian activity (La Marca & Volpe, 2006; Hazout *et al*., 2004). AMH shows less intra-individual fluctuation than AFC and basal FSH levels, and might be a better, cycle-independent parameter in assessing the ovarian reserve (Van Disseldorp *et al.*, 2010; Verhagen *et al*., 2008; La Marca & Volpe, 2006; La Marca *et al.*, 2013); however, AMH levels may decrease if measured during COS (Hamdine *et al.*, 2015).

The decrease in AMH levels that occurs with aging may be noted before changes in other age-related variables (La Marca *et al*., 2009), suggesting serum AMH levels may be a better marker of ovarian aging. AMH assays are being developed to demonstrate greater sensitivity, and they are likely to show greater value in this regard (Fleming *et al*., 2015).

In the last few years published studies have described the clinical application of AMH measurement in the prediction of quantitative and qualitative ovarian response in assisted reproductive technologies (ART). There is an association between AMH and oocyte yield after ovarian stimulation, and the hormone has been shown to be a strong predictor of ovarian response to gonadotropins, whether satisfactory, poor or excessive, cycle cancellation, and of the quality of oocytes and embryos (La Marca *et al*., 2005; Kavoussi *et al*., 2015).

A significant positive correlation has been described between serum AMH and the number of oocytes retrieved and mature oocytes. This correlation was considerably stronger than the associations found with other ovarian reserve markers such as serum FSH and estradiol (Seifer *et al*., 2002). Patients with undetectable AMH levels have been shown to successfully obtain oocytes at the time of retrieval and even to achieve ongoing pregnancy (Fraisse *et al*., 2008; Tokura *et al*., 2013). Therefore, a lower limit of AMH below which patients should not expect to have any ovarian response has not been established (Burks *et al*., 2015). A circulating AMH level of 0.7 ng/ml has been claimed to be the threshold value for poor ovarian response to controlled ovarian stimulation (COS), whereas levels below 0.1-0.35 ng/ml have been associated with high risk of cycle cancellation due to extremely poor response (Revelli *et al*., 2016).

## MATERIAL AND METHODS

This observational study looked into 287 antagonist ICSI cycles carried out with patients treated in a single center between January of 2012 and January of 2016. The maximum time interval between serum sampling and the start of COS was 12 months. Eighty per cent of the measurements were performed in the same laboratory. The following test kits were used to assess AMH levels: 2012 and 2013 - AMH Gen II ELISA (Beckman Coulter), an enzymatically amplified two-site immunoassay; 2014 - dual monoclonal antibodies in a chemiluminescent immunoassay (Quest Diagnostics); since 2015 - EletroChemiLuminescence, a technology developed by Roche for immunoassay detection (ELECSYS). The lower AMH detection limit was 0.012 ng/mL. The patients enrolled in the study consented to having their data discussed in scientific papers prior to the start of the cycles. Since this was a study based on data collected from patient charts, no further inquiries were made with the Ethics Committee.

Individual dosages were adjusted based on AMH levels. The patients were not pretreated with either oral contraceptives or estradiol. According to local protocol, stimulation began on day 2 or 3 of the cycle; most protocols used recombinant FSH (150-225 IU per day), with LH added for women ≥35 years (2:1 ratio FSH/ LH) and aromatase inhibitors whenever AMH <1 ng/mL (5mg per day, until the rhCG day). A daily GnRH antagonist dose of 0.25 mg was initiated based on a flexible protocol once a follicle ≥ 14 mm in diameter was seen in the ultrasound scan; antagonist therapy was continued until hCG administration. Gonadotropin doses could be adjusted from start of antagonist therapy. When at least one follicle was >18 and two follicles were ≥16mm in diameter, 250 mcg of recombinant human Chorionic Gonadotropin (Ovidrel^®^, Merck Serono SA.) was administered to induce final oocyte maturation. In patients at risk of OHSS, 0.2 mg triptorelin (Gonapeptyl^®^, Ferring Farmaceuticals) was used. Oocyte retrieval was performed under transvaginal ultrasound guidance 35 hours after ovulation induction.

The method we use in oocyte preparation and analysis has been described previously (Souza *et al*., 2009). This study focused solely on the prediction of metaphase II oocytes. Therefore, it included oocyte cryopreservation cycles, but fertilization, implantation and pregnancy rates on IVF/ICSI cycles were not addressed. Subgroup analyses were performed according to five AMH ranges: Group 1: ≤ 0.3 ng/mL (probably negligible response); Group 2: > 0.3 and ≤ 0.7 ng/mL (expected lower response); Group 3: > 0.7 and ≤ 1.0 ng/mL (possibly intermediate response); Group 4: > 1.0 and < 3.0 ng/mL (normal response); and Group 5: ≥ 3.0 ng/mL (high response). Observed variables included patient age; serum FSH and antral follicle count (AFC) at the start of the cycle; number of stimulation days; number follicles ≥ 15 mm on hCG day; number of oocytes retrieved and of metaphase II oocytes. Statistical analysis was performed by ANOVA. Significance was attributed to events with a *P*<0.05.

## RESULTS

The patients were divided into five groups based on the percentile ranks of AMH levels; the groups had 64, 76, 32, 86 and 29 patients, respectively. Canceled cycles, age, duration of stimulus, FSH levels, antral follicles, follicles ≥ 15 mm on hCG day, number of total oocytes, and number of metaphase II oocytes were found to be associated with AMH levels (Table 1).

**Table 1:** Parameters and ovarian stimulation outcomes according to AMH levels.

Parameter	Group 1 AMH ≤ 0.30	Group 2 > 0.30 AMH ≤ 0.70	Group 3 > 0.70 AMH ≤ 1.0	Group 4 > 1.0 AMH < 3.0	Group 5 AMH ≥ 3.0	*P*-value
Started cycles (287 total)	64	76	32	86	29	
BMI (kg/m^2^)	22.95 ± 2.77	23.95 ± 3.84	23.79 ± 3.41	23.23 ± 3.71	24.08 ± 3.97	NS
Age	37.83 ± 4.11^a^	38.61 ± 3.67^b^	38.15 ± 3.04^c^	37.23 ± 3.64	35.48 ± 3.97abc	^abc^<0.05
Serum FSH	12.45 ± 8.24^abcd^	8.95 ± 5.54^a^	7.99 ± 3.86^b^	7.40 ± 3.11^c^	6.81 ± 2.11^d^	^ab^<0.01 ^cd^<0.001
AFC	6.75 ± 3.47^ab^	9.11 ± 4.93^cde^	9.53 ± 3.74^f^	12.60 ± 5.24^acg^	17.58 ± 7.64^bdefg^	^abcefg^<0.001
Stimulation days	9.59 ± 1.73	9.41 ± 1.74	10.03 ± 1.53	9.24 ± 1.50	8.93 ± 1.10	NS
FSH administered (IU)	1673.66 ± 538.88	1744.57 ± 707.02	1805.64 ± 562.79	1725.72 ± 623.76	1620.27 ± 559.93	NS
Fol. ≥ 15 mm on hCG day	3.64 ± 2.26^abc^	5.36 ± 3.64^de^	6.34 ± 3.98^adf^	7.37 ± 3.60^beg^	11.13 ± 4.22^cfg^	^bcefg^<0.001 ^ad^0.01
No. of oocytes retrieved	3.27 ± 3.14^abc^	6.23 ± 6.85^ade^	5.87 ± 3.89^fg^	9.25 ± 5.10^bdfh^	16.96 ± 7.21^cegh^	^af^<0.05 ^d^<0.01 ^bcegh^<0.001
No. of metaphase II oocytes	1.89 ± 2.15^abc^	4.34 ± 5.96^ad^	4.09 ± 3.12^e^	6.30 ± 4.40^bf^	11.44 ± 6.35^cdef^	^a^<0.05 ^bcdef^<0.001

Data are presented as mean values (± standard deviation); AMH: Anti-Müllerian hormone (ng/mL);

FSH: Follicle Stimulating hormone; AFC: Antral Follicle Count; BMI: Body Mass Index;

hCG: human Chorionic Gonadotropin

There were no differences between groups in relation to the BMI. Female age was found to be an independent predictor of ovarian reserve. Statistical differences were found when groups 1, 2 and 3 were compared (mean age 38 years) to group 5 (age 35.48 years), *P* < 0.05. No differences were seen between group 4 (age 37.23 years) and the other groups.

The serum basal FSH levels of patients in the group with AMH levels ≤0.3 ng/mL were statistically different from the levels seen in the other groups. Indeed, poor responder groups differed mildly from each other but significantly when compared to normal or high responders.

No statistically significant difference was seen for antral follicle count (AFC) when groups 1 and 2 were compared (poorer prognosis groups) to group 3, but there was difference between groups 1 and 2 and groups 4 (*P* < 0.001) and 5 (*P* < 0.001). Interestingly, no difference was seen in AFC between groups 3 and 4 whereas all groups were statistically different from group 5 (*P* < 0.001).

In terms of duration of stimulus, no difference was found between groups, but the total amount of FSH required was significantly lower in group 1 ( ≤0.3 ng mL).

Another interesting finding was the statistical difference observed when groups 1 and 2 were compared to groups 3 and 4 for number of follicles ≥ 15 mm on hCG day (*P* < 0.01). Groups 1 and 2 had approximately 3 and 5 follicles ≥ 15 mm, respectively, while groups 3 and 4 had 6 and 7 follicles, respectively. No difference was seen when groups 3 and 4 were compared. However, there was a significant difference when the number of follicles seen in groups 1, 3 and 4 was compared to the number observed in group 5 (*P* < 0.001).

There was a significant difference in the total number of oocytes retrieved and metaphase II oocytes when groups 1 and 2 were compared (*P* < 0.05), as also seen when group 1 was compared to groups 4 and 5 (*P* < 0.001). No difference was observed between groups 2 and 3 in this regard. However, a difference was seen in the total number of oocytes retrieved when groups 2 and 3 were compared to groups 4 and 5. And, as expected, there was a statistical difference between groups 4 and 5 in this aspect. There was no statistical difference in the number of metaphase II oocytes between groups 2, 3 and 4. However, when these groups were compared to group 5, an expected statistically significant difference was verified (*P* < 0.001). No differences were observed between groups 1 and 3.

Table 2 shows treatment indications for all cases. Every group had patients willing to have their oocytes frozen, even when the prognosis was poor. Some patients had more than one treatment indication.

**Table 2:** Indication of treatment according group.

	Group 1	Group 2	Group 3	Group 4	Group 5
Ovarian factor	37*	38*	10	19	10*
Tubal	0	6**	3	15**	2
Endometriosis	6	2	4	8	3
Male	6*	7*/***	5	18***	6*
Unexplained	5	13	2	10	6
Social	1	2	0	1	2
Cryopreservation	8	9	8	12	2
Other	3	2	0	4	

* Associated male and ovarian /

** Associated male and tubal /

*** Associated male and endometriosis

AMH levels ≤0.70 (patients with poor prognosis) were observed in 140 patients (48.7%). Patients within this AMH level range accounted for 93% of the failed cycles (15 patients in group 1; 11 in group 2; one in group 3; and one in group 4). These numbers include cancelled cycles and patients with no oocytes or immature oocytes retrieved (Table 3).

**Table 3:** Cycle outcomes according to AMH levels.

	Initiated cycles	Cancelled cycles	Immature oocytes	No oocytes
Group 1 ≤ 0.3	64	8	3	3
Group 2 > 0.3 and ≤ 0.7	76	3	3	4
Group 3 > 0.7 and ≤ 1.0	32	0	1	0
Group 4 > 1.0 and ≤ 3.0	86	0	0	1
Group 5 > 3.0	29	0	0	0

## DISCUSSION

If AMH measurement is proposed to all women prior to the start of an IVF program, a clear definition of cutoff values for the prediction of poor and hyper-response is required to design treatment strategies. Nelson *et al.* (2009) also considered most of these aspects in a prospective study.

The main finding of this study was the association between AMH levels and retrieved metaphase II oocytes. AMH levels indicated that 48.7% of the cycles involved women with diminished ovarian reserves, and 13.6% of the patients were good candidates for cryopreservation. This information confirms the need of good counseling when it comes to tailoring the stimulation protocol. Much effort has been made to identify patients with good prognosis based on AMH levels (Souza *et al.*, 2014), as La Marca *et al*. (2010), Hamdine *et al.* (2015), and others have pointed out.

A statistically significant difference in mean serum AMH levels and age of female patients was identified for AMH levels ≤0.7 when compared to other groups. This is a relevant point in our practice, as 40% of our patients are aged 38 years or older. And the same applies to the Latin American Registry, in which 70% of the patients are aged 35 years and older (Zegers-Hochschild *et al.*, 2016).

La Marca *et al.* (2010) predicted normal response for individuals with AMH levels ≥ 0.66 and < 1.99 ng/mL and high response for subjects with AMH levels ≥ 1.99 ng/mL. The AMH levels of our group of normal responders ranged from 1 to 3 ng/mL, while high responders (at risk to OHSS) had AMH levels above 3 ng/mL.

Our failed stimulations situated mainly in the groups with AMH levels ≤0.7 ng/mL, and oocytes were retrieved even with very low levels. Reichman *et al*. (2014) also found, after studying 2,760 patients and 4,072 cycles, that AMH positively correlates with the number of oocytes retrieved and is a robust predictor of COS IVF cycle failure.

One of the drawbacks of our AMH level measurement protocol (Iliodromiti *et al*., 2014; Rustamov *et al*., 2014) is that we had three different assays used during the course of the study. Assays have become more robust with time and AFC values remained stable, as the protocol was performed in the same center and using the same technique. In this study, patients with poorer prognosis had statistically different AFC and basal FSH levels when compared to women in good prognosis groups (normal and high responders). AFC alone did not yield significant differences between the three groups with poorer prognosis.

Different indications for ART in the different AMH level groups might explain the cycles without transfer (Table 2). Patients with lower AMH levels underwent IVF/ICSI treatment mostly because of a detected reduced ovarian reserve. The indication of ART for patients with high AMH levels was usually based on the presence of anovulatory cycles due to PCOS. The group with normal AMH levels included mainly ovulatory women with other IVF/ICSI indications such as male factor, tubal factor or unexplained infertility, and endometriosis, as described by other authors (Gomez *et al*., 2015).

The present study strongly supports previously published papers discussing the prognostic value of AMH levels upon total number of oocytes and oocyte quality. A possible limitation in this study is the time interval between serum sampling and start of COS. However, it is rather unlikely that such a time interval has impacted our results, as a time interval up to 12 months between serum sampling and initiation of stimulation has been shown not to affect the predictive ability of AMH (Polyzos *et al*., 2013).

Anti-Müllerian hormone allows for better selection and individualization of stimulation protocols, and should be added to the toolkit of assisted reproduction physicians as indicated by Fleming *et al.*, 2015. Our study found a significant positive correlation between serum AMH levels and the number of oocytes retrieved and mature oocytes. AMH levels were a considerably stronger marker of ovarian reserve than serum FSH, as also reported by Seifer *et al*. (2002).

Patients with undetectable AMH levels have had oocytes retrieved and have been able to achieve ongoing pregnancy (Fraisse *et al*., 2008; Tokura *et al.*, 2013). Therefore, a lower limit of AMH below which patients should not expect to have any ovarian response has not been established (Burks *et al*., 2015). A circulating AMH level ≤ 0.7 ng/mL deserves attention for having poor ovarian response to COS and presenting a higher risk of cycle failure.

## CONCLUSION

AMH is particularly useful to predict the quality of ovarian response to stimulation, independently from patient age. Women with AMH levels ≥1.0 and ≤3.0 ng/mL are probably normal responders with good prognosis. Clinical application depends on individual centers examining their own data, correlating AMH levels and ultimate ovarian response in the form of metaphase II oocytes.
